# The Influence of Ecological and Conventional Plant Production Systems on Soil Microbial Quality under Hops (*Humulus lupulus*)

**DOI:** 10.3390/ijms15069907

**Published:** 2014-06-03

**Authors:** Karolina Oszust, Magdalena Frąc, Agata Gryta, Nina Bilińska

**Affiliations:** Institute of Agrophysics, Polish Academy of Sciences, P.O. BOX 201, Lublin 20-290, Poland; E-Mails: koszust@ipan.lublin.pl (K.O.); agryta@ipan.lublin.pl (A.G.); nbilinska@ipan.lublin.pl (N.B.)

**Keywords:** diversity, organic farming, soil microbial functionality

## Abstract

The knowledge about microorganisms—activity and diversity under hop production is still limited. We assumed that, different systems of hop production (within the same soil and climatic conditions) significantly influence on the composition of soil microbial populations and its functional activity (metabolic potential). Therefore, we compared a set of soil microbial properties in the field experiment of two hop production systems (a) ecological based on the use of probiotic preparations and organic fertilization (b) conventional—with the use of chemical pesticides and mineral fertilizers. Soil analyses included following microbial properties: The total number microorganisms, a bunch of soil enzyme activities, the catabolic potential was also assessed following Biolog EcoPlates^®^. Moreover, the abundance of ammonia-oxidizing archaea (AOA) was characterized by terminal restriction fragment length polymorphism analysis (T-RFLP) of PCR ammonia monooxygenase α-subunit (*amo*A) gene products. Conventional and ecological systems of hop production were able to affect soil microbial state in different seasonal manner. Favorable effect on soil microbial activity met under ecological, was more probably due to livestock-based manure and fermented plant extracts application. No negative influence on conventional hopyard soil was revealed. Both type of production fulfilled fertilizing demands. Under ecological production it was due to livestock-based manure fertilizers and fermented plant extracts application.

## 1. Introduction

Scientific research in the field of soil microbiology serves inter alia for environment protection and ecological farming, and thus constitutes an important and fast-growing branch of science in recent years. Ecological hop production currently makes up a small but steadily increasing percentage of the worldwide hops supply [1]. As new farms are established to grow food ecologically, new solutions are still being searched to fulfill all standards of ecological farming. It is expected that these solutions would be a valuable alternative choice for traditional farms as far as the quality and volume of production is concerned, together with its economic profitability. The interest in this subject follows worldwide agriculture development trends, specified by The Food and Agriculture Organization of the United Nations (FAO) policy and European Committee objectives, including environment conservation and protection against degradation, increase in the matter sources and soil productivity improvement. Furthermore, according to European Union Council Regulation (EEC) No. 2092/91 (Annex I, 2.4) [2], preparations of microorganisms are admitted for application in organic farming in order to improve the overall condition of the soil or the availability of nutrients in the soil or in the crops.

Literature mentions how ecological farming using organic amendment, affects biological soil properties [3,4]. Although there are limited research results concerning the influence of an ecological farming, based on probiotic additives and organic matter application, on soil microbial quality in hop production. Since, the substantial benefits, namely improvement in quality and the increasing amounts of many crops after probiotic preparations application were claimed, farmers have started to invest in. At the same time they expect to have the best method of crop cultivation. Nowadays, farmers are still being suggested and persuaded that addition of positive microorganisms inoculants changes the soil microbial community towards dominance of beneficial species, and thus suppresses harmful bacteria, which produce toxic compounds. However, there is not much known about the effectiveness and practical consequences of these additives [5]. It seems important to determine the significance of probiotic inoculants introduced into soil in relation to functional and genetic diversity of soil microbial communities, comparing to the microbial state of soils under conventional farm production system. Microbial inoculants are expected for potential of improving efficiency of N_2_-fixation, nutrient availability to plants, prevention for infection by phytopathogens as biocontrol agents [6].

Assessing the abundance of microorganisms is often tested as a part of environment monitoring system, together with soil respiratory and enzymatic activity and the intensity of processes vital for soil fertility, that is carbon and nitrogen circulation [7]. These tests are used to determine soil fertility and productivity and they also make it possible to examine and understand the complexity of changes taking place in the soil environment [8]. Additionally, the implementation of molecular biology techniques into soil analysis allows for a very probable determination of microorganisms community contents and its metabolic dynamics in a given soil environment. It is also possible to determine and define the main role of the dominants performing different kinds of metabolic process [9,10,11]. Genetic diversity analysis of microorganisms communities [12] and the description of changes in the metabolome of environmental samples both complement the traditional, enzymatic diagnostic techniques used to study changes in the microbiological condition of soil under different factors.

The knowledge on microorganisms diversity is crucial to understand the relationships between environmental parameters and the function of ecosystem [7,9]. Microorganisms diversity is extremely important because of their role in mineralization processes and in providing nutrients, which is closely connected with a decline in intensive farming and, in consequence, a decrease in the use of mineral fertilisers and pesticides. Microorganisms diversity greatly influences the upkeep of balance in the soil functioning, and the changes in microorganisms strains structure are an important element of soil quality monitoring [13,14].

Presented study has important cognitive significance, because current state of knowledge is fragmentary in the field of biodiversity of microorganisms populations occurring at hop roots. It is also unclear what is the nature of relations between microorganisms introduced into soil and those microorganisms which had already been there. Therefore, it is crucial elucidate the relations between plants and microorganisms in the root zone, to understand the mechanisms which maintain biodiversity and keep agricultural ecosystems in a healthy state.

## 2. Results

**Figure 1:**
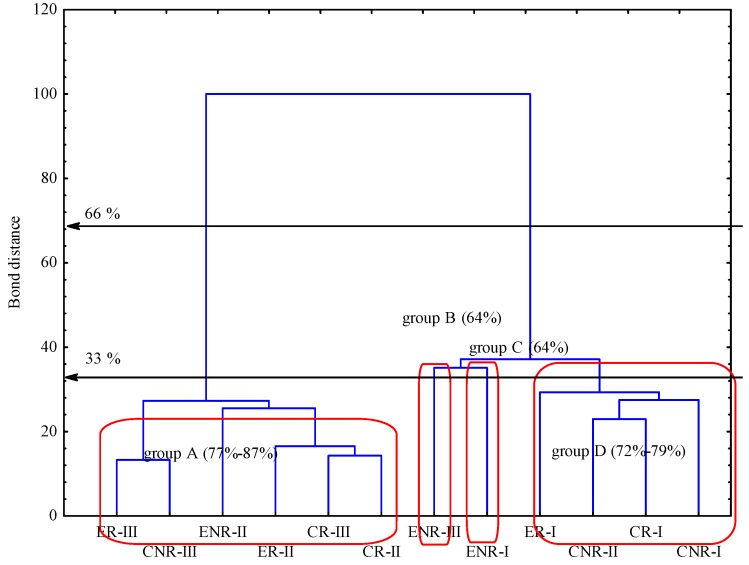
Dendogram of carbon utilizations patterns of substrates located on Biolog EcoPlates^®^. Grouping according to the stringent Sneath criterion (33%), and less restrictive criterion (66%), respectively. Explanations: CR, conventional rhizosphere soil; CNR, conventional non-rhizosphere soil; ER, ecological rhizosphere soil; ENR, ecological non-rhizosphere soil; I, II, III phases of hop vegetation, subsequently called terms of analyses. Vertical error bars represent the standard error of the mean; *n* = 3.

Table 1 presents biodiversity indices of soil microbial communities catabolic potential average well colour development (AWCD) index showed that rhizosphere soils of conventional and ecological production (CR and ER, respectively) in I term revealed relatively similar average metabolic response, whereas dynamics change of AWCD in II and III terms differed, namely in improving AWCD in ER. However, when non-rhizosphere soils taken under consideration, we observed the same tendency in both treatments, but at much lower AWCD values in ecological non-rhizosphere soil (ENR). Richness index (*R*) was consistent to AWCD. Oppositely, shannon biodiversity index (*H*) showed generally that objects did not differ as far as microbial functional community stability within the EcoPlate^®^ incubation time, but with the exception of conventional rhizosphere soil in I term. Dendogram (Figure 1) presenting similarity of carbon utilizations patterns of substrates located on Biolog EcoPlates^®^. Taking into account the stringent Sneath criterion (33%) there are four similar groups distinguishable. Regarding less restrictive criterion (66%) the number of similar groups is merely two. Based on 33% Sneath criterion objects are gathered into four groups (A–D) where obtained values of C-substrate utilization are in the range of 77%–89%, 64%, 64%, 72%–79%, from A to D respectively. Such a type of obtained gathering was due to the most utilized carbon substrate, which were Tween 80; d-Lactose, Phentylthylamine and d-Mannitol in group A. Groups B and C were independent with d-Mannitol, Erythritol and α-Cyclodextrin as the most intensively utilized substrates. Group D was separated mostly on the basis of utilizing d-xylose and l-Asparagine.

**Figure 2:**
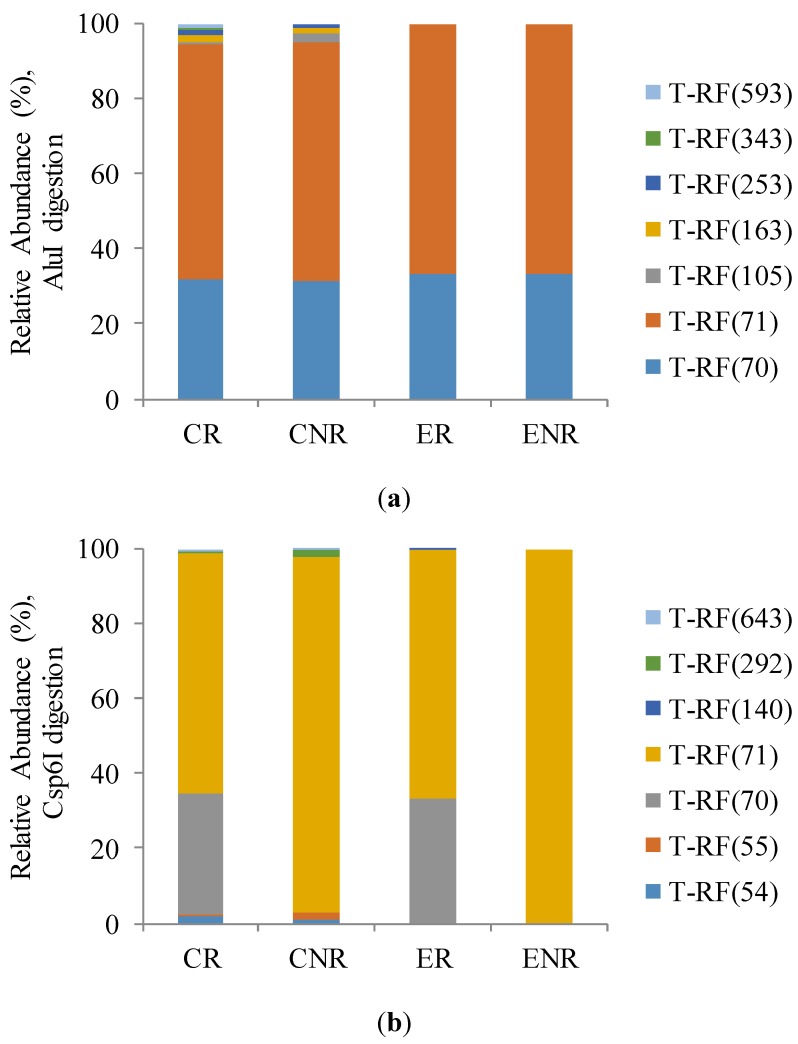
Relative abundance (%) of ammonia-oxidizing archaea (AOA) *amo*A gene sequences fragments (T-RFs) after (**a**) AluI and (**b**) Csp6I digestion.

The AOA terminal restriction fragment length polymorphism (T-RFLP) profiles (Figure 2a,b) showed that there was only two major T-RFs of 70 and 71 bp corresponding in both independent restriction profiles with the tetrameric restriction enzymes AluI and Csp6I, among 7 operational taxonomic units (OTUs) revealed. The remaining 5 OTUs were different for AOA T-RFLP profiles after digestion with AluI and Csp6I. Although, its relative abundance was lower than 3% they differed objects clearly with greater differentiation in conventional production system than under ecological one. Restriction process with Csp6I enzyme revealed higher ammonia-oxidizing archaea diversity in soil under conventional production.

**Table 1:** Biodiversity indices of soil microbial communities catabolic potential.

Term	Treatment	AWCD	*R*	*H*
I	CNR	13.64 ± 0.90 ^bcd^	22.66 ± 1.15 ^cde^	3.24 ± 0.03 ^abc^
	CR	11.39 ± 1.26 ^cd^	18.00 ± 1.73 ^de^	3.07 ± 0.09 ^cd^
	ENR	8.48 ± 1.72 ^d^	16.00 ± 4.58 ^e^	3.03 ± 0.27 ^d^
	ER	13.44 ± 1.44 ^bcd^	23.00 ± 5.00 ^bcde^	3.25 ± 0.07 ^abc^
II	CNR	12.99 ± 2.01 ^bcd^	23.67 ± 2.08 ^abcd^	3.25 ± 0.08 ^abc^
	CR	17.45 ± 2.54 ^ab^	30.33 ± 1.15 ^ab^	3.38 ± 0.02 ^a^
	ENR	16.27 ± 1.18 ^abc^	28.00 ± 1.00 ^abc^	3.36 ± 0.03 ^a^
	ER	19.81 ± 2.59 ^a^	30.67 ± 0.58 ^a^	3.39 ± 0.01 ^a^
III	CNR	17.11 ± 0.28 ^ab^	28.33 ± 1.53 ^abc^	3.34 ± 0.01 ^a^
	CR	19.06 ± 2.93 ^a^	30.33 ± 0.57 ^ab^	3.37 ± 0.04 ^a^
	ENR	12.94 ± 1.67 ^bcd^	24.00 ± 4.00 ^abcd^	3.28 ± 0.04 ^abc^
	ER	16.19 ± 1.71 ^abc^	28.67 ± 1.53 ^abc^	3.35 ± 0.02 ^a^

±, standard deviations for three replications. Different letters within the same variables indicate significant differences (*p* < 0.05). Explanations: CR, conventional rhizosphere soil; CNR, conventional non-rhizosphere soil; ER, ecological rhizosphere soil; ENR, ecological non-rhizosphere soil; I, II, III phases of hop vegetation, subsequently called terms of analyses; AWCD, average well-color development; *R*, richness; *H*, Shannon-Weaver index.

Seasonal variations were observed in enzymatic activity of soil samples from conventional and ecological hop production systems. Furthermore, we noticed significant differences in rhizosphere and non-rhizosphere soil among a bunch of tested enzymatic activities (Table 2a,b). As shown in Table 2a ammonification rate (AMO), as well as protease activity (PA), and both alkaline and acidic phosphatases (ALP and ACP) were found to be the most exuberant in rhizosphere soil under ecological production (ER) comparing to the other treatments. Ammonification rate was the highest in ER in I and II term; ACP in ER in I and III term, ALP in II term, whereas protease activity in both rhizosphere and non-rhizosphere soil (ER and ENR respectively) was the highest in III term. Urease (URE) and respiratory activity (RESP), presented Table 2b, were higher under conventional production compared to ecological production system. Urease activity in non-rhizosphere soil under conventional production (CNR) exceeded ecological one in I term of analyses. As well as respiratory activity in III term of CR and CNR soils was 42% and 23% respectively, higher than under ecological treatment. However, as far as remaining types of examined activities (nitrification, dehydrogenases and β-glucosidase), shown in Table 2b, these properties cannot be clearly attributed to production system, because of differences in sampling term and soil root zone. Nitrification rate tended to be higher in II term in CR and in III term under ER than in other treatments. We reported β-glucosidase activity higher in III term of CR, and in II term of CNR. It was proved that dehydrogenases activity in II term was highest CR and ENR and in III term in both rhizosphere and non-rhizosphere soil of conventional production. Annual means of evaluated enzymatic activities are presented in Table 2a,b. Ecological rhizosphere (ER) fostered significantly average of acid phosphatase activity, intense of ammonification and protease activity and reduced respiratory activity (non-significant).

Table 2Enzymatic activity. (**a**) Ammonification rate, protease activity, acidic and alkaline phosphatase activity; and (**b**) Urease activity, respiratory activity, nitrification rate, dehydrogenases activity and β-glucosidase activity.

**(a):** 

Term	Treatment	AMO	PA	ACP	ALP
		(mg N–NH_4_ kg^−1^)	(mg tyrosine kg^−1^·h^−1^)	(mmol PNP kg^−1^·h^−1^)	(mmol PNP kg^−1^·h^−1^)
I	CR	0.00 ± 0.00 ^b^	7.56 ± 0.16 ^b^	19.9 ± 11.1 ^c^	5.35 ± 2.86 ^de^
	CNR	52.4 ± 13.1 ^ab^	6.69 ± 0.52 ^b^	58.0 ± 16.9 ^ab^	6.07 ± 2.38 ^de^
	ER	115 ± 7.51 ^a^	10.4 ± 2.17 ^b^	47.3 ± 25.0 ^abc^	0.00 ± 0.00 ^e^
	ENR	38.3 ± 36.0 ^ab^	12.2 ± 0.38 ^b^	56.4 ± 0.00 ^ab^	0.00 ± 0.00 ^e^
II	CR	33.9 ± 3.30 ^ab^	7.56 ± 0.16 ^b^	31.8 ± 9.56 ^abc^	8.09 ± 2.06 ^cde^
	CNR	0.00 ± 0.00 ^b^	6.69 ± 0.52 ^b^	33.5 ± 2.36 ^abc^	13.3 ± 3.32 ^abcd^
	ER	98.2 ± 8.09 ^a^	10.4 ± 2.17 ^b^	25.9 ± 2.15 ^bc^	20.5 ± 3.27 ^ab^
	ENR	2.48 ± 0.00 ^b^	12.2 ± 0.38 ^b^	46.1 ± 16.5 ^abc^	9.22 ± 2.78 ^cde^
III	CR	94.4 ± 83.2 ^a^	50.0 ± 16.3 ^ab^	42.4 ± 2.30 ^abc^	16.2 ± 4.06 ^abc^
	CNR	36.9 ± 18.6 ^ab^	22.3 ± 1.40 ^b^	29.1 ± 1.08 ^bc^	20.7 ± 2.58 ^ab^
	ER	64.7 ± 14.6 ^ab^	105 ± 56.87 ^a^	62.4 ± 0.63 ^a^	20.5 ± 1.80 ^ab^
	ENR	77.1 ± 17.5 ^ab^	64.8 ± 45.7 ^ab^	44.2 ± 3.14 ^abc^	24.1 ± 7.32 ^a^
**Annual means**					
	CNR	29.8 ± 26.0 ^b^	11.9 ± 7.86 ^a^	40.2 ± 16.0 ^a^	13.4 ± 6.78 ^a^
	CR	42.8 ± 58.7 ^ab^	21.7 ± 22.7 ^a^	31.4 ± 12.2 ^a^	9.87 ± 5.56 ^a^
	ENR	38.4 ± 38.9 ^b^	29.7 ± 34.8 ^a^	48.9 ± 10.1 ^a^	11.1 ± 11.2 ^a^
	ER	92.7 ± 24.1 ^a^	41.8 ± 55.0 ^a^	45.2 ± 20.2 ^a^	13.7 ± 10.4 ^a^

±, standard deviations for three replications. Different letters within the same variables indicate significant differences (*p* < 0.05). Explanations: CR, conventional rhizosphere soil; CNR, conventional non-rhizosphere soil; ER, ecological rhizosphere soil; ENR, ecological non-rhizosphere soil; I, II, III phases of hop vegetation, subsequently called terms of analyses; AMO, intense of ammonification; PA, protease activity; ACP, acidic phosphatase activity; ALP, alkaline phosphatase activity.

**(b):** 

Term	Treatment	URE	RESP	NITR	DHA	BGL
		(mg N–NH_4_ kg^−1^·h^−1^)	(mg CO_2_ kg^−1^·day^−1^)	(mg N–NO_3_ kg^−1^)	(mg TPF kg^−1^·day^−1^)	(mg PNG kg^−1^·h^−1^)
I	CR	1.82 ± 0.75 ^ab^	122 ± 12.2 ^e^	249 ± 2.04 ^abc^	0.58 ± 0.14 ^e^	1.21 ± 0.59 ^cde^
	CNR	4.25 ± 1.44 ^a^	119 ± 6.28 ^e^	292 ± 1.48 ^a^	0.43 ± 0.06 ^e^	1.02 ± 0.15 ^de^
	ER	1.32 ± 0.36 ^ab^	142 ± 6.18 ^de^	260 ± 27.31 ^ab^	1.54 ± 0.15 ^e^	0.87 ± 0.23 ^de^
	ENR	1.46 ± 0.39 ^ab^	121 ± 19.0 ^e^	216 ± 9.63 ^bcd^	0.09 ± 0.05 ^e^	0.19 ± 0.04 ^e^
II	CR	0.29 ± 0.09 ^b^	150 ± 6.00 ^cde^	156 ± 33.4 ^def^	8.51 ± 0.19 ^a^	1.20 ± 0.58 ^cde^
	CNR	1.46 ± 1.78 ^ab^	151 ± 18.1 ^cde^	103.9 ± 56.0 ^efgh^	4.04 ± 0.20 ^d^	2.14 ± 0.32 ^bc^
	ER	0.59 ± 0.28 ^b^	138 ± 5.99 ^de^	56.0 ± 48.9 ^gh^	4.71 ± 1.32 ^cd^	2.78 ± 0.74 ^ab^
	ENR	1.82 ± 2.93 ^ab^	175 ± 5.65 ^bc^	51.5 ± 7.90 ^gh^	7.24 ± 1.36 ^ab^	0.76 ± 0.16 ^de^
III	CR	0.74 ± 0.00 ^b^	265 ± 12.1 ^a^	37.2 ± 3.59 ^h^	6.44 ± 1.37 ^abc^	3.49 ± 0.35 ^a^
	CNR	0.55 ± 0.44 ^b^	267 ± 12.1 ^a^	84.6 ± 13.1 ^fgh^	5.94 ± 0.40 ^bcd^	0.37 ± 0.05 ^de^
	ER	0.95 ± 0.73 ^b^	157 ± 12.1 ^cd^	176 ± 7.80 ^cde^	1.19 ± 0.45 ^e^	1.40 ± 0.30 ^de^
	ENR	1.06 ± 0.50 ^ab^	200 ± 6.04 ^b^	123 ± 5.39 ^efg^	1.02 ± 0.16 ^e^	0.35 ± 0.02 ^de^
**Annual means**						
	CNR	2.09 ± 2.03 ^a^	179 ± 68.3 ^a^	160 ± 103 ^a^	3.47 ± 2.43 ^a^	1.18 ± 0.79 ^a^
	CR	0.95 ± 0.77 ^a^	179 ± 66.4 ^a^	147 ± 93.3 ^a^	5.18 ± 3.63 ^a^	1.97 ± 1.22 ^a^
	ENR	1.45 ± 1.53 ^a^	165 ± 36.5 ^a^	130 ± 1.70 ^a^	2.78 ± 3.43 ^a^	0.43 ± 0.27 ^a^
	ER	0.95 ± 0.53 ^a^	146 ± 11.5 ^a^	164 ± 94.3 ^a^	2.48 ± 1.82 ^a^	1.68 ± 0.94 ^a^

±, standard deviations for three replications. Different letters within the same variables indicate significant differences (*p* < 0.05). Explanations: CR, conventional rhizosphere soil; CNR, conventional non-rhizosphere soil; ER, ecological rhizosphere soil; ENR, ecological non-rhizosphere soil; I, II, III phases of hop vegetation, subsequently called terms of analyses; URE, urease activity; RESP, respiratory activity; NITR, nitrification rate; DHA, dehydrogenases activity; BGL, β-glucosidase activity.

It was found that total number of bacteria (TNB), *Bacillus*, and *Pseudomonas* (TN*Bac* and TN*Pseud*, respectively) and fungi (TNF) exceeded in ecological production as presented in Table 3. Regardless the term, they were mostly found in rhizosphere soil. On the other hand it was noted that they exceeded mostly in non-rhizosphere soil and differed when term taken into consideration. This was as follows: TNB was the highest in CNR in I and III term and in I and II term in ER. We noticed more *Pseudomonas* spp. colony-forming units in II term of CNR than in corresponding microcosm of ecological production (ENR). When ER taken into consideration II term was abounded in *Pseudomonas* spp. TN*Bac* was highest in CNR II term. Table 3 also presents annual means of total number of particular groups of microorganism. Most of tested group are more numerous in conventional than ecological non-rhizosphere soil. As far as rhizosphere soil more abundant in microorganisms is ecological one.

**Table 3:** Total number of microorganisms.

Term	Treatment	TNF	TNB	TN *Bac*	TN *Pseud*
		(CFU 10^6^ kg^−1^)	(CFU 10^6^ kg^−1^)	(CFU 10^6^ kg^−1^)	(CFU 10^6^ kg^−1^)
I	CR	15.5 ± 1.92 ^e^	3.27 ± 1.89 ^d^	11.4 ± 0.53 ^bcd^	17.1 ± 5.59 ^b^
	CNR	21.3 ± 4.62 ^e^	23.7 ± 6.04 ^bc^	17.4 ± 5.73 ^bcd^	17.4 ± 5.73 ^b^
	ER	41.6 ± 7.03 ^cd^	24.3 ± 1.26 ^bc^	37.5 ± 17.2 ^bcd^	30.0 ± 6.49 ^b^
	ENR	26.9 ± 3.33 ^cd^	5.49 ± 1.1 ^d^	23.0 ± 1.00 ^bcd^	23.1 ± 11.5 ^b^
II	CR	14.9 ± 3.15 ^e^	3.27 ± 1.89 ^d^	32.9 ± 12.2 ^bcd^	17.2 ± 4.14 ^b^
	CNR	159 ± 6.70 ^a^	23.7 ± 6.04 ^bc^	73.2 ± 29.31 ^a^	65.2 ± 21.8 ^a^
	ER	203 ± 35.6 ^a^	24.3 ± 1.26 ^bc^	44.7 ± 8.10 ^abc^	70.7 ± 6.02 ^b^
	ENR	48.4 ± 13.9 ^cd^	5.49 ± 1.10 ^d^	3.29 ± 3.80 ^e^	2.19 ± 1.90 ^b^
III	CR	110 ± 32.4 ^b^	33.6 ± 8.37 ^bc^	48.2 ± 10.0 ^ab^	70.8 ± 7.69 ^a^
	CNR	55.2 ± 8.82 ^cd^	23.5 ± 11.1 ^bc^	32.4 ± 10.4 ^bcd^	30.9 ± 5.83 ^b^
	ER	67.4 ± 5.64 ^bc^	36.4 ± 0.91 ^a^	45.1 ± 6.68 ^abc^	80.2 ± 14.0 ^a^
	ENR	11.4 ± 7.15 ^e^	20.3 ± 4.33 ^bc^	9.52 ± 2.28 ^e^	27.5 ± 11.2 ^b^
**Annual means**					
	CNR	78.5 ± 71.7 ^ab^	23.7 ± 0.12 ^ab^	41.0 ± 28.9 ^a^	37.8 ± 24.6 ^ab^
	CR	46.6 ± 34.5 ^ab^	13.4 ± 7.51 ^bc^	30.8 ± 18.5 ^b^	35.0 ± 31.0 ^ab^
	ENR	28.9 ± 18.6 ^b^	10.4 ± 8.54 ^c^	12.0 ± 10.1 ^b^	17.6 ± 13.5 ^b^
	ER	104 ± 86.7 ^a^	28.4 ± 6.97 ^a^	42.4 ± 4.26 ^a^	60.3 ± 26.7 ^a^

±, standard deviations for three replications. Different letters within the same variables indicate significant differences (*p* < 0.05). Explanations: CR, conventional rhizosphere soil; CNR, conventional non-rhizosphere soil; ER, ecological rhizosphere soil; ENR, ecological non-rhizosphere soil; I, II, III phases of hop vegetation, subsequently called terms of analyses; TNF, Total number of fungi; TNB, the number of bacteria; TN*Bac*, Total number of *Bacillus*; TN*Pseud*, the number of *Pseduomonas*.

**Table 4:** Correlation coefficients (*r*) between examined microbial parameters.

Parameters	TNF	TNB	TN *Bac*	TN *Pseud*
**TNB**	* 0.41			
**TN *Bac***	*** 0.63	** 0.46		
**TN *Pseud***	*** 0.72	*** 0.72	*** 0.63	
**DHA**	n.s.	n.s.	n.s.	n.s.
**PA**	n.s.	** 0.48	n.s.	* 0.40
**URE**	n.s.	n.s.	n.s.	n.s.
**ACP**	n.s.	n.s.	n.s.	n.s.
**ALP**	n.s.	** 0.51	n.s.	** 0.46
**AMO**	n.s.	** 0.47	n.s.	* 0.36
**NITR**	*** −0.55	n.s.	n.s.	* −0.36
**RESP**	n.s.	* 0.37	n.s.	n.s.
**BGL**	*** 0.72	** 0.43	** 0.52	*** 0.63

*, **, ***, indicated significance at the *p* < 0.05, *p* < 0.01, and *p* < 0.001 level, respectively; n.s., no significant; TNB, total number of bacteria; TN*Bac*, total number of *Bacillus*; TN*Pseud*, total number of *Pseudomonas*; DHA, dehydrogenase activity; PA, protease activity; URE, urease activity; ACP, acid phosphatase; ALP, alkaline phosphatase; AMO, ammonification activity; NITR, nitrification activity; RESP, respiratory activity; BGL, β-glucosidase activity.

TNF was highly (*p* < 0.001) correlated to nitrification and β-glucosidase activities (negative correlation). TNB was correlated to protease, alkaline phosphatase, ammonification and β-glucosidase activities (all mentioned with the positive correlation). TN*Bac* and TN*Pseud* were also correlated to β-glucosidase. Furthermore, TN*Pseud* was noted to be correlated at lover layer *p* < 0.05 with ammonification, alkaline phosphatase and nitrification activity. TN*Bac,* TN*Pseud*, TNB, TNF were highly, positively correlated to each other (Table 4).

## 3. Discussion

The evidence proposed by Hole *et al.* [15] indicates that soil microbial communities are likely to be affected by farming regime as much as by edaphic factors. In our experiment different systems of hop production (within the same soil and climatic conditions) were able to alter soil microbial state, however in different time manner. This albeit seasonal fluctuations, apparently do not led to meaningful differences in quantity of hop yields (data not shown). Nevertheless, wide appraisal of potential role of main production systems’ components in soil microbial biodiversity conservation in hops agroecosystems was made.

As Mayer *et al.* [6] suggested, under field conditions in ecological production it might be expected that the inoculated microorganisms establish an indigenous microbial community in the soil after repeated applications and a longer application period and/or stimulate the indigenous microorganisms and processes. Initial effects of this inoculants may first be indicated by a change of soil microbial parameters. Evidently, when biochemical parameters and the number of microorganisms taken into consideration we found this favorable effect under ecological production. On the other hand, the data draws attention to community catabolic potential followed carbon substrates utilization pattern, located on Biolog EcoPlates^®^. Both conventional and ecological rhizosphere soil, regardless term of analysis, were quite similar. It was due to similar functional profiles in microbial communities occurred, because of the root exudates effect [16]. Whereas conventional and ecological non-rhizosphere soils grouped separately, indicating different microbial metabolic profiles of inherent communities, where the reach of mentioned substances was limited. However, this effect could be doubted to reflect reality of the functional abilities of the entire soil microbial community, as the Community level physiological profiles (CLPP) approach is a cultivation based method and is able to discriminate treatments with bias towards populations, growing under assay condition [17,18]. Therefore, we followed the need, stressed by Ros *et al*. [19] for a multi-parameter approach, when examining the impact of production management on soil condition, and we accompanied CLPP method with other, inter alia structural diversity investigations.

In the course of this microbial processes going in the rhizosphere it is also interesting how different production systems can affect nitrogen cycling. Nitrification, which results in the formation of NO_3_^−^^1^, is carried out by a few specialists. Relatively recent reports revealed that archaea predominate among ammonia-oxidizing prokaryotes in soils, not bacteria, as it was said [20]. We followed this reports in our experiments evaluating an abundance of ammonia-oxidizing archaea (AOA) T-RFLP profiles in total community DNA. Based on Csp6l digestion we revealed higher ammonia-oxidizing archaea diversity soil under conventional production (second term) compering to ecological one. Greater biodiversity of AOA met under conventional production compering to nitrification level under ecological production were demonstrating consistency. What is more, surprisingly we also noted significantly higher intensity of ammonification in ecological rhizosphere in II term. As seasonal variations occurred, we looked into detail comparing *amo*A AOA T-RFLP profiles and corresponding enzyme activity engaged in nitrification process. Its comparable annual averages clearly suggest that soil environment regulates its microbial biodiversity inherently. It is said that compost and manure have the advantage that useable nitrogen is released more slowly.

Nevertheless, Turner *et al.* [1] suggest, high levels of N should be available before periods of rapid bine growth begins. Though little N is absorbed (about 10%) before mid-June, by the end of July, hops have generally taken up the majority of the annual N, between 90 and 180 kg N·ha^−1^. The challenge meet crop N demands is time limited of mineralization and release of N from biofertilizers during crucial uptake periods. In our experiment we did not note any significant differences between conventional and ecological soils. What is more, the most intense nitrifying activity was met in the first term of analysis. Thus, we assumed that both type of production fulfilled N demands presented above. Therefore, microbial activity and biodiversity in soil under hopyards play the substantial role. It is not clear if all the positive effect under ecological production was supported by microorganisms consisting on probiotic inoculants per se, but as Mayer *et al*. [6] claim, specific etcetera’s (fermentable organic substrate—Bokashi or even sugar cane molasses, ethanol or vinegar) included in such as preparations. Furthermore, Schweinsberg-Mickan and Müller [21] suggested exogenous organic matter application as a biofertilizer might have been a reason for affirmative results. Also Okorski and Majchrzak [22] found an increase in the abundance of fungal species in the rhizosphere of peas and a similar diversity in a field experiment if those inoculants were applied, compared to the unamended control. Thus, horse manure in our experiment was probably the main factor stimulating microbial properties. Systems that receive high organic matter (OM) inputs have greater labile C pools, greater microbial activity and greater soil N supplying power compared to systems that receive only mineral fertilizer [23]. Less is known about the actual rates of short-term microbial N transformations in systems that differ in C availability and soil N supplying capacity [24].

Seasonal variations of microbial soil properties, we observed under both production systems, may result from a “priming effect” of short-term change in the turnover of soil organic matter (SOM) induced either by addition of compounds to the soil or by soil disturbance [25]. The author suggests that a positive priming effect occurs when easily available root exudates increase the activity and density of rhizosphere microorganisms, which is consistent with our findings in ecological rhizosphere soil. Instead, root-induced changes in SOM decomposition rate may also affect nutrient cycling because they influence nutrient availability and thereby nutrient uptake by plants. Such a situation could occur in conventional soil. Negative priming effects may was found as having an inhibitory effect on microorganisms activity and abundance.

It address the issue that, conventional plant production system, chemical crop protection products and mineral fertilisers used, may disturb natural biological processes, and consequently, cause a disorder in the ecosystem balance. It can also contribute to a decrease of the beneficial saprophytic microflora and a limitation of soil self-purification abilities [8].

Congruent with this statement are our results of lower abundance of beneficial microorganisms such as *Bacillus* and *Pseudomonas* groups in conventional rhizosphere (CR), pointing at more favorable conditions provided in soil by ecological production, especially when biotic interactions between microorganisms in the rhizosphere may have their impact on the plant growth, development and health eventually, as it is suggested in literature [26]. Surprisingly, the same effect was not met in non-rhizosphere soil. In non-rhizosphere soil under ecological production (ER) we observed, oppositely to the rhizosphere soil, firmly reduced number of colony forming units of *Bacillus* and *Pseudomonas*. However, it cannot be clearly confirmed that simultaneous effect of higher total number of bacteria and fungi in ecological rhizosphere soil is righteous upshot of ecological treatment. The used analyses do not reflect—the proportion of beneficial microorganisms (plant growth—promoting rhizobacteria, PGPR), and harmful microorganisms (deleterious rhizosphere microorganisms, DRMO). Nevertheless improper proportion of this groups may eventually influence plant growth in long term farming. Even though, the evidence are clear that microbial communities are likely to be affected by production systems.

In opposite to microbial abundance we revealed, that in both soils under—ecological and conventional systems self-purification conditions, based on soil biochemical properties, are quite similar. Thus we did not observe significant differences in annual average of soil enzymatic activities. However the seasonal shifts are very forcible in here. Most of those examined activities did not differ non-rhizosphere soil of conventional and ecological productions. Nonetheless, ecological rhizosphere (ER) fostered significantly average of acid phosphatase activity, intense of ammonification and protease activity showed its tendency to reduce respiratory activity (non-significant). Predominance of phosphatase activity in ecological (ER) and lower in conventional rhizosphere (CR) can be explained by the depletion of soil organic phosphorus in CR due to the only use of inorganic forms of phosphorus in conventional [27].

## 4. Methodology

### 4.1. Experimental Set-up

The field experiment was established on an eutric cambisol soil type developed from loess, in Jastków near Lublin, Poland (22°27'E, 51°19'N). Experimental fields comprised hop plantations (Marynka variety), cultivated in two treatments, as follows:

Ecological plant production system—cultivation in the manner set out in the Act on ecological farming of 25 June 2009;

(1) 6 year of vegetation, cultivated ecologically, and 3 year of fertilization with horse manure 20 mg·ha^−1^ and EM-Farming™ preparations;

(2) The treatment with the use of probiotic microorganisms was carried out based on ProBioEms preparations, included the list of ecological farming products: EM-Farma Plus (Certificate NE/71/2006) and EMa5 (attestation PZH/HT 2052/2006), EMa5 with tansy (Tanacetum) coming from maternal element ProBioOriginal;

Conventional plant production system—cultivation in the manner set out in the Act on plant protection of 18 December 2003; treatment with mineral fertilisers and chemical crop protection products.

Each plantations (108 plants) comprising three rows (36 plants in each) was additionally divided into three plots (3 rows of 12 plants). Samples of hops root soil were taken by randomizing approach and included:

Rhizosphere soil (1–5 mm from the external root surface); the soil zone (concerned as a growing medium) in which the environment for microbial activity is influenced by any root growing in it directly. Treatments were as follows: CR, conventional rhizosphere soil; ER, ecological rhizosphere soil.

Non-rhizosphere soil—“bulk”—the soil zone, which is influenced by growing roots by water and nutrients withdrawal [28]. Rhizosphere and non-rhizosphere soil was collected from the soil profile surface layer, *i.e*., 0–20 cm deep, from every of the above mentioned experimental treatments. The depth of the soil samples was due to the fact, that this was the hops root system zone which was directly exposed to contact with the ingredients present in the probiotic preparations used in the field experiment. Treatments were as follows: CNR—conventional non-rhizosphere soil, ENR—ecological non-rhizosphere soil.

#### 4.1.1. The Collection and Preparation of the Non-Rhizosphere Soil for the Research.

To reduce the amount of fresh organic carbon getting into the soil, the plant layer was removed, together with the visible parts of roots and parts of plants and also soil fauna. Soil samples for the evaluation of genetic profile differences in the soil microbial communities (for genomic DNA extraction) was collected into specially labelled 50 mL tubes with caps, by screwing the test tubes was gently into soil, so that soil fills up 30 mL of a test tube. Soil samples for the evaluation of differences in the soil metabolic condition was collected from the same layer in each experimental field, next they was put into specially labelled polyethylene bags. The test tubes and bags with the collected samples was placed in heat-insulating bags and transported to the laboratory. In the laboratory the soil was thoroughly blended and sifted through 2 mm sieves. Such prepared samples were used to draw the necessary amount of soil for further analysis.

#### 4.1.2. The Collection and Preparation of the Rhizosphere Soil

Soil samples for the research were collected in three subsequent phases of hops vegetation, subsequently called as terms: I—before rootstock was formed, II—during bloom phase, III—after cone harvest. Roots with a lump of earth was collected from 10 randomly chosen spots on each experimental field. The soil, sticking to the roots (1–5 mm), was shaken off and put into plastic test tubes or polyethylene bags, and serve as rhizosphere. Laboratory preparation—the same procedure as in the above mentioned non-rhizosphere soil samples preparation.

### 4.2. Community Level Physiological Profiling

Community Level Physiological Profiling followed Biolog EcoPlate^®^ (Biolog Inc., Hayward, CA, USA) with a set of carbon sources proposed by Insam [29]. Each well of the Biolog EcoPlate^®^ was inoculated with 120 μL of inoculum and incubated at 27 °C. Absorbance readings were taken periodically (every 24 h for 168 h for each soil sample) at 590 nm with a plate reader Biolog MicroStation™. Moreover, Richness index (*R*), Shannon (*H*), and average well colour development index (AWCD) were calculated following Garland and Millis [30].

### 4.3. Terminal Restriction Fragment Length Polymorphism Analysis (T-RFLP) Analysis of Ammonia-Oxidizing Archaea (AOA)

Abundance of ammonia-oxidizing archaea (AOA) in total community DNA of II term under both treatments was characterized by T-RFLP analysis of *amo*A PCR products ammonia monooxygenase α-subunit (*amo*A) gene. Genomic DNA was extracted from 0.5 g from every object using a FastDNA^®^ SPIN Kit for Faeces (MP Biomedicals, Solon, OH, USA) following the producer protocol. The amount of DNA was determined by Spectrophotometer (NanoDrop 2000/2000c Thermo Scientific, West Palm Beach, FL, USA) at 260 nm. The PCR was performed in a total volume of 30 µL containing 4 ng of DNA template, 15 μL RedTaq^®^ ReadyMix™ PCR Reaction Mix (Sigma-Aldrich, St. Louis, MO, USA). The primers used for PCR were 0.5 µM and were as follows: Starter F: 6-carboxyfluorescein-FAM 5'-ATGGTCTGGCTWAGACG-3' starter R: 5'-TCCCACTTWGACCARGCGGCCATCCA-3' [20,31]. Thermal cycling was carried out by an initial denaturation step at 95 °C for 5 min. The major cycling program for each primer set was optimized and was as listed: (92 °C, 45 s; 59 °C, 30 s; 72 °C, 60 s) × 35; 72 °C, 7 min. The presence and sizes of the PCR amplification products (700 bp) were determined by agarose (1.3%) gel electrophoresis. The PCR products were purified by using ExoSAP-IT^®^ PCR Products Purification Kit for ABI (Affymetrix Inc., Santa Clara, CA, USA) followed with incubation at 37 °C for 15 min and then 15 min at 80 °C. The restriction mixture (10 µL), containing 7 µL of purified PCR product (about 50 ng DNA), 0.6 µL of buffer Tango (Fermentas^®^ International, Burlington, ON, Canada), and 0.6 µL of restriction enzyme (10 U/µL) AluI or Csp6I (Fermentas^®^ International, Burlington, ON, Canada), respectively, was incubated at 37 °C for 2 h. The reaction was stopped by incubation at 65 °C for 20 min. Aliquots (1 µL) of the digest were mixed with 9 µL deionized formamide and 0.5 µL DNA fragment length standard (GS-600LIZ, ABI) (Applied Biosystems, Foster City, CA, USA). The mixture was denatured at 94 °C for 3 min and snap-cooled on ice. The fluorescently labelled T-RFs were run through an ABI 3130 xl capillary sequencer (Applied Biosystems, Foster City, CA, USA) in the GeneScan mode. T-RFLP data was analysed using GeneMaper^®^ Software v4.0 (Applied Biosystems, Foster City, CA, USA). Because of the detection range of internal marker Lys, T-RFs smaller than 50 bp and larger than 643 bp were excluded from further analysis. The relative abundance of each T-RF was determined by calculating the ratio between the area of each peak and the total area of all peaks in one sample. The peaks with relative abundance <1% were neglected in this study.

### 4.4. Total Numbers of Culturable Microorganisms

Total number of bacteria (TNB) was determined with the plate method on a medium with soil extract and K_2_HPO_4_, number of *Bacillus* spp*.* (TN*Bac*) on Trypticasein Soy Lab-Agar (Biocorp, Warsaw, Poland), number of *Pseudomonas* spp. (TN*Pseud*) on Pseudomonas F Lab-Agar (Biocorp, Warsaw, Poland). Total number of fungi (TNF) was determined using Martin’s Rose Bengal Lab-Agar (Biocorp, Warsaw, Poland) with antibiotics Streptomycine and Chlorotetracycline addition. For each microbiological analysis three replicates per treatment were done.

### 4.5. Enzymatic Activity

Dehydrogenases activity (DHA) was determined according to Thalmann [32], after soil incubation with 2,3,5-triphenyl-tetrazolium chloride (TTC) and measuring the triphenyl formazan (TPF) absorbance at 485 nm. Protease activity (PA) was determined by the Ladd and Butler method [33], with Alef and Nannipieri [34] later modifications, followed measurement of the concentration of tyrosine released by soil after 1 h incubation at 50 °C with a Tris–HCl (pH 8.1) casein solution. The tyrosine concentration was measured at 578 nm. Alkaline and acidic phosphatases (ALP and ACP, respectively) were determined according to Tabatabai and Bremner method [35], after soil incubation with *p*-nitrophenyl phosphate disodium and measuring the *p*-nitrophenol (PNF) absorbance at 400 nm. The respiratory activity (RESP) was determined by substrate-induced respiratory according to Rühling and Tyler [36] method. Ten milligram glucose g^−1^ dry soil was added to obtain maximum initial respiratory response. A method of assaying urease activity (URE) in soils was used as described by Zantua and Bremner [37]. β-Glucosidase activity (BGL) was assessed with *p*-nitrophenyl-β-d-glucoside (PNPG) solution as a substrate (25 mM) based on method evaluated by Eivazi and Tabatabai [38]. Ammonification activity (AMO) and intense of nitrification process (NITR) were assessed according to PN-ISO 15685:2007P [39].

### 4.6. Statistical Analysis

The microbial properties results were investigated statistically. All statistical analyses were performed with Statistica 10.0 software (StatSoft Inc., Tulsa, OK, USA, 2011). Analysis of variance (two-way ANOVA) and mean comparisons between treatments was used with Tukey’s *post hoc* honestly significant differences (HSD) at *p* < 0.05. Differences in 7 AOA OUTs (operational taxonomic units) were detected among treatments. Relative abundance (in %) of each AOA OUT was determined by corresponding normalized T-RFLP area. Cluster analysis including grouping of treatments and features was performed on standardized data of absorbance average values for readings of 120 h. Dendogram presenting similarity of carbon utilizations patterns of substrates located on Biolog EcoPlate^®^, between soil samples was set on scaled axis bond distances (Ward’s method), with marked boundaries Sneath’s criteria (33% and 66%) was prepared.

## 5. Conclusions

Conventional and ecological systems of hop production (within the same soil and climatic conditions) were able to affect soil microbial state in different seasonal manner.

Apparently this fluctuations, do not led to meaningful differences in quantity of hop yields. However, crop N demands is time limited of mineralization and release of N from biofertilizers during crucial uptake periods, we assumed that both type of production fulfilled N demands presented in literature.

Despite the seasonal differences in maximum enzymatic activity and microbial abundance the soil environment regulated its microbial biodiversity inherently, whereas significant differences between annual means of tested microbial parameters in ecological and conventional production were not found.

Favorable effect of ecological production on soil microbial activity, was presumably due to livestock-based manure fertilizers and fermented plant extracts application, rather than microbial inoculants activity.
